# Mental health challenges of lesbian, gay, bisexual and transgender people: An integrated literature review

**DOI:** 10.4102/hsag.v26i0.1487

**Published:** 2021-01-20

**Authors:** Miriam M. Moagi, Anna E. van Der Wath, Priscilla M. Jiyane, Richard S. Rikhotso

**Affiliations:** 1 Department of Nursing Science, Faculty of Health Sciences, University of Pretoria, Tshwane, South Africa

**Keywords:** LGBT, mental health disparities, discrimination, stigmatisation, victimisation

## Abstract

Lesbian, gay, bisexual and transgender (LGBT) individuals are often stigmatised and discriminated against. This population is expected to experience poorer mental health outcomes compared with heterosexual and cis-gendered people, a phenomenon healthcare providers need to take note of and act upon. This study aimed to explore and describe the mental health challenges of LGBT people. An integrative literature search was conducted. The following electronic databases were searched: Academic Search Premier, Africa-Wide Information, Business Source Premier, eBook Collection (EBSCOhost), E-Journals, ERIC, Family & Society Studies Worldwide, Health Source: Nursing/Academic Edition, Humanities Source, MasterFILE Premier, MEDLINE, PsycARTICLES, Social Work Abstracts, TOC Premier, WorldCat.org, Taylor and Francis Journals, Biomed Central and Wiley Online Library. An internet search was also carried out using Google and Google Scholar databases. The following search terms were identified: ‘LGBT’ OR ‘LGBT community’ AND ‘mental health challenges/problems’ OR ‘mental/psychiatric illness’. The reviewed literature comprised research conducted globally between 2010 and 2019. From the 2545 titles, 345 abstracts were examined, resulting in 57 articles. The 57 full-text articles were examined to verify whether they addressed the scope of the literature review, of them, 21 addressed the mental health challenges of LGBT people. Lesbian, gay, bisexual and transgender people experience the following mental health challenges: emotional distress, stigmatisation, victimisation, discrimination and barriers to accessing healthcare services. The results showed that although LGBT has been legalised in many countries, LGBT communities still experience significant mental health challenges. Healthcare providers are in a position to address challenges related to social and healthcare structures and act as advocates in order to promote the mental health of LGBT individuals.

## Introduction

The acronym LGBTQ (also LGBTQAP+, LGBTQA, GLBTIQ, LGBT, LGBTQ and other alternates) is an umbrella term that stands for lesbian, gay, bisexual, transgender, intersex, and queer or questioning people. This acronym has its origins in the shorter version lesbian, gay, bisexual and transgender (LGBT), which covers a heterogeneous group of LGBT people who often feature together as a group in efforts to gain better social representation and more political support (Salminen 2015:11). Although the term LGBT has its restrictions and does not cover all possible identities and orientations (Salminen 2015:11), it is used in this article to denote all people who belong to sexual and gender minorities.

The LGBT community was historically marginalised, mistreated and ignored by society and healthcare delivery systems (Farmer & Yancu 2015:36; Institute of Medicine 2011:1). This community faces various obstacles to gain access to quality healthcare (Duby et al. 2018:8; Sequeira, Chakraborti & Panunti 2012:379) and experiences poorer health and mental health outcomes compared with heterosexual and cis-gendered (gender identity matches the sex assigned at birth) people (Cochran & Mays 2007:2048; Farmer & Yancu 2015:41). Understanding the mental health needs of sexual minorities and the causes of mental health disparities is a rapidly growing area of research, especially regarding mental health outcomes with implications for policies (Mongelli et al. 2019:47). This article presents the results of an integrative literature review with regards to the mental health challenges experienced by LGBT people.

Mental health is fundamental to appropriate psychological processes, healthy relationships and living a fulfilled life. The promotion, protection and restoration of mental health are vital to individuals, communities and societies throughout the world. Programmes targeted at vulnerable people, including minorities, are seen as ways to strengthen worldwide responses to mental health (World Health Organization 2018).

The LGBT people face significant social and legal barriers; in 76 countries, same-sex sexual acts are still criminalised with penalties that can include fines, several years of imprisonment or even execution (United Nations Programme on HIV/AIDS 2013:4). For example, in Ukraine, LGBT people who wish to be legally recognised must undergo a compulsory, psychiatric evaluation to confirm or reject a diagnosis of ‘transsexualism’. Some transgender people are arrested by police who sometimes sexually abuse them under the pretext of cleaning up public spaces (Ghoshal & Knight 2011:24). In other countries, such as South Africa, the constitution protects every person irrespective of sexual orientation. This assurance occurs in various human rights accords and confirms the self-respect and self-worth of each person, including LGBT individuals (Constitution of the Republic of South Africa 1996: Section 9).

The acronym LGBT combines sexual orientation with gender identity. Sexual orientation is the ‘enduring emotional, romantic, sexual or affectional attraction to another person’ (American Psychological Association 2008:1). For gay men, this attraction is primarily to men, and for lesbians, this is primarily to the woman. Gender identity is a person’s self-perception as a man or woman (Farmer & Yancu 2015:37). Transgender refers to people whose gender identity is at odds with the gender they were assigned at birth according to their sex and physiological characteristics (Fredriksen-Goldsen et al. 2014:3). Sexuality encompasses at least three key components: sexual identity, sexual attraction and sexual behaviour. Sexual identity refers to the cognitive and emotional meaning one attaches to expressions of sexuality (Farmer & Yancu 2015:37), which includes romantic, emotional and social preferences (Morgan 2013:53). Sexual orientation, sexual identity and gender identity are not static and may change over a person’s life course. The LGBT individuals have unique experiences that are shaped by multiple factors, such as race/ethnicity, socio-economic status, geographical location and age, not just sexual orientation (Farmer & Yancu 2015:37).

The lesbian, gay, bisexual and transgender people differ from ‘traditional’ minorities in two aspects: (1) they are not necessarily recognisable through physical characteristics and (2) they are still perceived in many contexts as acting against natural processes (Takács 2015:10). These people suffer from various forms of socio-economic and cultural injustices, but mostly they feel they are denied recognition, meaning that heterosexuality is privileged and homosexuality is devaluated (Takács 2015:9). In order to fully understand the challenges the LGBT community faces, it is important to understand the concept of heteronormativity, which is still accepted in many segments of society and refers to the ‘normalisation of heterosexuality through social structures, social practices, and social institutions’ (Javaid 2018:84). The belief that other sexual orientations are abnormal or inferior to heterosexuality is a source of oppression, resulting in heterosexism and homophobic attitudes, creating a hostile climate for LGBT people (Mostert, Gordon & Kriegler 2015:116; Salminen 2015:11).

## Aims

This integrative literature review aimed to review the current literature and to explore and describe the mental health challenges of LGBT people.

## Design

**FIGURE 1:**
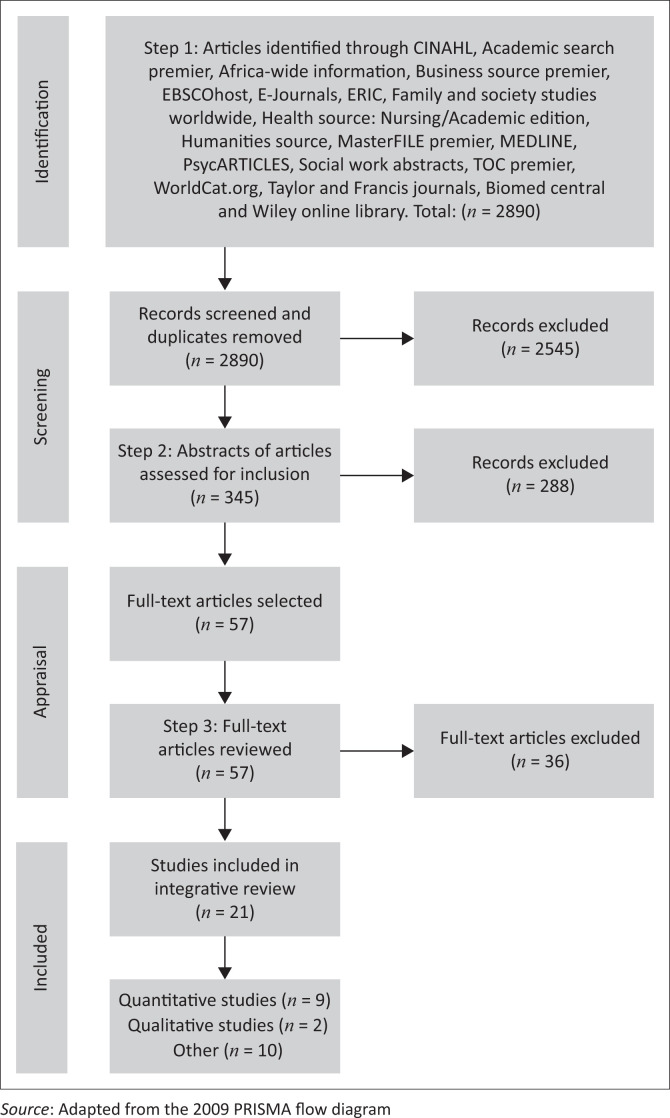
PRISMA flow diagram describing the inclusion process of the integrative literature review.

An integrative review method as proposed by Whittemore and Knafl (2005:547–549) was used, as it allowed for the inclusion of diverse methodologies (experimental and non-experimental research) to explore different perspectives on the mental health challenges of LGBT people. The following four stages were followed: literature search, data evaluation, data analysis and presentation of findings (see Figure 1).

## Methods

### Literature search

A literature search was performed according to the following inclusion criteria: quantitative or qualitative research or reports from experts and different organisations or institutions with a vast interest in the mental health challenges of the LGBT community, published between 2010 and 2019 in English (translation cost was too high for publications in other languages). Letters, editorials and commentaries were excluded as the review focused on research findings and expert reports on the topic. In order to increase the robustness of the search, the authors requested an experienced librarian to review the literature for inclusion or exclusion criteria and suggest databases and websites include in the search. The following search terms were identified: ‘LGBT’, OR ‘LGBT community’ AND ‘mental health challenges/problems’, OR ‘mental/psychiatric illness’.

Given the fact that LGBT is a social, political, health and economic problem; an electronic search was launched through the Cumulative Index of Nursing and Allied Health Literature (CINAHL), using the databases Academic Search Premier, Africa-Wide Information, Business Source Premier, eBook Collection (EBSCOhost), E-Journals, ERIC, Family & Society Studies Worldwide, Health Source: Nursing/Academic Edition, Humanities Source, MasterFILE Premier, MEDLINE, PsycARTICLES, Social Work Abstracts, TOC Premier, WorldCat.org, Taylor and Francis Journals, Biomed Central and Wiley Online Library. An internet search was also carried out using Google and Google Scholar databases to find reports from experts and different organisations/institutions with interest in the mental health challenges of the LGBT community. The search results were initially broad and were narrowed using more specific search topics. The reference lists from retrieved studies were manually searched.

The literature search as described above resulted in 2890 citations. The titles of these publications were screened for potential relevance. The researchers excluded 2545 publications based on the inclusion and exclusion criteria. Duplicated publications were also excluded. The abstracts of the remaining 345 publications were assessed by two researchers who are experienced in mental health nursing. The researchers analysed the abstracts to select publications directly related to the mental health challenges experienced by LGBT people. Using the stipulated criteria, a further 288 documents were excluded. After a critical appraisal of the full text of the remaining 57 publications, 36 more publications were excluded, which were not describing the mental health challenges experienced by LGBT people, but focused on other aspects, for example, mental health challenges experienced by people diagnosed with a bipolar mood disorder. Twenty-one publications remained within the review, which was relevant to the mental health challenges experienced by LGBT people.

### Data evaluation

The final 21 selected publications consisted of theoretical and empirical reports. All publications were tabulated according to the author(s), year of publication, country, design and method, population and sampling, and purpose. Owing to the diverse representation of sources, six criteria evaluating methodological quality (modified based on Kangasniemi, Pakkanen & Korhonen 2015:1746; Whittemore & Knafl 2005:549–550) were used to evaluate the publications on a three-point scale as ‘high’, ‘low’ or ‘not reported’ (Table 1). The majority of the empirical reports followed a quantitative approach where data were collected through surveys, whilst two studies used qualitative methods. In three publications, the authors based the discussion on a conceptualisation or briefing of different theoretical approaches. Three studies followed a longitudinal or retrospective design. One study piloted a mental health programme, and five publications provided literature or historical overview.

**TABLE 1:** Evaluation of publications.

Author(s) Year Country	Design and method Population and sample	Purpose	Quality appraisal (scale: h, high, l, low, nr, not reported)
Blosnich and Andersen (2015) USA	Quantitative survey. *N* = 20.060 noninstitutionalised adults over the age of 18, probability-based sample from USA states and territories and the District of Columbia	To test the hypothesis that adverse childhood experiences explain the association between LGB status and mental distress amongst adults.	(h) Aims and objectives clearly stated (h) Study design adequately described (h) Research methods appropriate (nr) Explicit theoretical framework (h) Limitations presented (h) Implications discussed
Fredriksen-Goldsen et al. (2014) USA	Conceptualisation	To present the Health Equity promotion model (oriented towards LGBT people to reach their full mental and physical health potential and considers positive and adverse health-related circumstances)	(h) Aims and objectives clearly stated (nr) Study design adequately described (nr) Research methods appropriate (h) Explicit theoretical framework (nr) Limitations presented (h) Implications discussed
Hatzenbuehler et al. (2013) USA	Conceptual framework	To provide evidence on the health consequences of stigma and present a conceptual framework describing psychological and structural pathways through which stigma influences health.	(h) Aims and objectives clearly stated (nr) Study design adequately described (nr) Research methods appropriate (h) Explicit theoretical framework (nr) Limitations presented (h) Implications discussed
Hatzenbuehler et al. (2014) USA	Quantitative survey. *N* = 914 subjects who had sexual partners of the same sex in the past 12 months, the past 5 years, or since age 18, representative sample of sexual minorities from the non-institutionalised English-speaking USA population aged 18 and over.	To investigate whether structural stigma (living in communities with high levels of anti-gay prejudice) increases the risk of premature mortality for sexual minorities.	(h) Aims and objectives clearly stated (h) Study design adequately described (h) Research methods appropriate (h) Explicit theoretical framework (h) Limitations presented (h) Implications discussed
Heck (2015) USA	Pilot study. *N* = 10 members of a high school gay-straight alliance in the north-eastern United States	To determine the feasibility and acceptability of a mental health promotion programme to address minority stressors and promote coping skills amongst LGBTQ youth.	(h) Aims and objectives clearly stated (h) Study design adequately described (h) Research methods appropriate (h) Explicit theoretical framework (h) Limitations presented (h) Implications discussed
Institute of Medicine (2011) USA	Consensus method. *N* = 17 experts in the fields of mental health, biostatistics, clinical medicine, adolescent health and devel-opment, ageing, parenting, behavioural sciences, HIV research, demography, racial and ethnic disparities and healthcare services.	To review and assess the state of the science on the health status of LGBT populations. To identify research gaps and opportunities. To outline a research agenda.	(h) Aims and objectives clearly stated (h) Study design adequately described (h) Research methods appropriate (h) Explicit theoretical framework (h) Limitations presented (h) Implications discussed
Kerr et al. (2013) USA	Secondary comparative analysis of quantitative health assessment data. *N* = 6689 self-identified lesbian, bisexual and heterosexual female college students who took part in the assessment of American College Health Association National College Health Assessment data set for three semesters.	To investigate selected mental health characteristics of lesbian and bisexual undergraduate college women when compared with heterosexual college women.	(h) Aims and objectives clearly stated (h) Study design adequately described (h) Research methods appropriate (nr) Explicit theoretical framework (h) Limitations presented (h) Implications discussed
Mallory et al. (2017) USA	Report compiled from the literature.	To document the prevalence of several forms of stigmatisation and discrimination against LGBT adults and youth in Texas.	(h) Aims and objectives clearly stated (nr) Study design adequately described (nr) Research methods appropriate (nr) Explicit theoretical framework (nr) Limitations presented (h) Implications discussed
McCann and Sharek (2014) Ireland	Qualitative design. *N* = 20 people aged 18 and older who identified as LGBT, and had used Irish mental healthcare services in the past 5 years participated in semi-structured interviews.	To explore the experiences and needs of LGBT people in relation to mental healthcare services.	(h) Aims and objectives clearly stated (h) Study design adequately described (h) Research methods appropriate (nr) Explicit theoretical framework (h) Limitations presented (h) Implications discussed
Mongelli et al. (2019) Italy	Systematic review	To summarise the recent literature on the relationship between minority stress experienced by sexual minorities and mental health.	(h) Aims and objectives clearly stated (h) Study design adequately described (h) Research methods appropriate (h) Explicit theoretical framework (h) Limitations presented (h) Implications discussed
Mustanski et al. (2016) USA	Longitudinal study data collected in seven waves over 4 years using structured psychiatric interviews. *N* = 248 participants who identified as LGBT or report the same-sex attractions from the Chicago, Illinois area.	To examine the effects of the cumulative victimisation experienced by LGBT youths on mental disorders.	(h) Aims and objectives clearly stated (h) Study design adequately described (h) Research methods appropriate (nr) Explicit theoretical framework (h) Limitations presented (h) Implications discussed
Ojanen et al. (2016) Thailand,	Historical review	To give a historical overview of LGBT identities and issues; highlight psychiatric, psychological and nursing research on LGBT mental health and services; and review LGBT-related policy of organisations dealing with mental health in Thailand.	(h) Aims and objectives clearly stated (nr) Study design adequately described (nr) Research methods appropriate (nr) Explicit theoretical framework (nr) Limitations presented (h) Implications discussed
Ojeda-Leitner and Lewis (2019) USA	Online survey. *N* = 95 members of the LGBT community, 18 years or older.	To explore the impact of health-related stereotype threats and its influence within a LGBT sample	(h) Aims and objectives clearly stated (h) Study design adequately described (nr) Research methods appropriate (nr) Explicit theoretical framework (h) Limitations presented (h) Implications discussed
Robles et al. (2016) Mexico	Retrospective structured interview design. *N* = 250 transgender adults, 18 years or older, purposively sampled and receiving healthcare services at a clinic in Mexico.	To determine whether distress and impairment could be explained by experiences of social rejection and violence rather than being inherent features of transgender identity. To examine the applicability of other elements of the ICD-11 diagnostic guidelines.	(h) Aims and objectives clearly stated (h) Study design adequately described (h) Research methods appropriate (nr) Explicit theoretical framework (h) Limitations presented (h) Implications discussed
Rosenstreich (2011) Australia	Briefing paper	To consider discrimination as a key determinant of disproportionately poor mental health outcomes amongst LGBTI people and outline implications for mental healthcare services.	(h) Aims and objectives clearly stated (nr) Study design adequately described (nr) Research methods appropriate (nr) Explicit theoretical framework (nr) Limitations presented (h) Implications discussed
Russell and Fish (2016) USA	Literature review	To provide an overview of the contemporary context for LGBT youth and of current science on LGBT youth mental health, focusing on theoretical and empirical foundations. To consider the state of knowledge of risk and protective factors, specific to LGBT youth and their experiences and those that are amenable to change through prevention or intervention.	(h) Aims and objectives clearly stated (nr) Study design adequately described (h) Research methods appropriate (h) Explicit theoretical framework (nr) Limitations presented (h) Implications discussed
Rutherford et al. (2012) Canada	Descriptive phenomenological design using semi-structured interviews. *N* = 8 participants from four professional disciplines: psychiatry, social work, psychotherapy and psychology.	To explore how providers with LGBT-focused practices have developed their capacity for working with these populations.	(h) Aims and objectives clearly stated (h) Study design adequately described (h) Research methods appropriate (h) Explicit theoretical framework (h) Limitations presented (h) Implications discussed
Steele et al. (2017) Canada.	Cross-sectional internet survey. *N* = 704 sexual and gender minority people and heterosexual cis-gendered adult women target sampled across Ontario, Canada.	To compare the past year unmet need for mental healthcare and untreated depression between four groups: heterosexual cisgender (i.e. not transgender) women, cisgender lesbians, cisgender bisexual women and transgender people.	(h) Aims and objectives clearly stated (h) Study design adequately described (h) Research methods appropriate (nr) Explicit theoretical framework (h) Limitations presented (h) Implications discussed
Su et al. (2016) USA	Online survey. *N* = 770 respondents who self-identified as lesbian, gay, bisexual and/or transgender (91 transgender and 676 non-transgender), over the age of 19 in Nebraska.	To assess whether transgender identity is associated with an elevated probability of reported discrimination, depression symptoms and suicidal attempts compared with non-transgender LGB individuals. To determine whether LGBT identity acceptance is associated with a lower probability of depression symptoms in transgender and non-transgender LGB individuals.	(h) Aims and objectives clearly stated (h) Study design adequately described (h) Research methods appropriate (nr) Explicit theoretical framework (h) Limitations presented (h) Implications discussed
Utama (2017) Indonesia	Literature review	To describe the four major mental health issues (depression, anxiety, substance use disorder and suicide) and to explore its determinants amongst LGBTI persons in Indonesia.	(h) Aims and objectives clearly stated (h) Study design adequately described (h) Research methods appropriate (h) Explicit theoretical framework (h) Limitations presented (h) Implications discussed
Lozano-Verduzco et al. (2017) Mexico	Cross-sectional study using face-to-face questioning and a digital survey tool. *N* = 2846 LGBT individuals non-probabilistic and intentionally sampled in Mexico.	To analyse the association between internalised homophobia, homophobic violence, discrimination, and community connectedness and alcohol use and depressive symptoms in LGBT individuals	(h) Aims and objectives clearly stated (h) Study design adequately described (h) Research methods appropriate (nr) Explicit theoretical framework (l) Limitations presented (i) Implications discussed

*Source:* Adapted from Kangasniemi, M., Pakkanen, P. & Korhonen, A., 2015, ‘Professional ethics in nursing: An integrative review’, *Journal of Advanced Nursing* 71(8), 1744–1757. https://doi.org/10.1111/jan.12619

LGBTQ, lesbian, gay, bisexual, transgender and queer; LGBTI, lesbian, gay, bisexual, transgender and intersexual; LGBT, lesbian, gay, bisexual and transgender; LGB, lesbian, gay and bisexual.

### Data abstraction and synthesis

Two researchers analysed the selected publications independently by thematic analysis. Key results or meanings related to the research aim were highlighted in the publication and summarised and listed. The lists of key results or meanings were clustered according to themes and sub-themes. The researchers reached a consensus on the themes and sub-themes to describe mental health challenges of LGBT people. The results from the data analysis are displayed in Table 2. The results are presented as three main themes, and each theme is presented under the following headings: sub-themes, key findings and sources (author, year and country).

**TABLE 2:** Results of the integrative review.

Sub-themes	Key results	Sources
**Theme 1: Emotional distress as a mental health challenge**		
**Incidence of emotional distress**	*LGB individuals:* Significantly higher mean totals of adverse childhood experiences and a significantly greater proportion of reported mental distress than in heterosexual individuals. LGB status not significantly associated with mental distress. Adverse childhood experience significantly associated with mental distress. Social and structural determinants contribute to sexual orientation-based mental health disparities. *LGBT individuals:* Higher rates of depression, suicidality and substance use. Face numerous mental health disparities. *LGB youth:* Increased risk for suicidal ideation and attempts and depression. *LGB adults:* Experience more mood and anxiety disorders, and an elevated risk for suicidal ideation and attempts compared with heterosexual adults. *Bisexual women:* Reported the worst mental health status in anxiety, anger, depressive symptoms, self-injury, and suicidal ideation and attempts. Bisexual women and lesbians: Greater likelihood of having mental health issues than heterosexual women. Utilised significantly more mental healthcare services than heterosexual women. The study population (undergraduate students) had free access to counselling.	Blosnich and Andersen (2015), USA Mongelli et al. (2019) Italy Institute of Medicine (2011) USA Kerr et al. (2013) USA
**Minority stressors**	*LGBT youth:* Mental health risk factors related to structural/societal levels and interpersonal interactions with family and peers characterised by minority stress. Need for studies to identify intrapersonal strengths or coping strategies in order to overcome minority stress. *LGBT individuals:* Internalised homophobia. Expectation of rejection. Identity concealment. *LGBTQ individuals:* Experience of prejudice events. Expectation to experience prejudice events or rejection. Engagement in strategies to conceal LGBTQ status. Internalisation of negative societal views of LGBTQ status. Experience of discrimination/victimisation (prejudice event). Expectation of rejection and discrimination. Concealment of sexual orientation. Internalisation of homophobia. Levels of minority stressors positively predict mental health outcomes.	Russell and Fish (2016) USA Fredriksen-Goldsen et al. (2014) USA Heck (2015) USA Mongelli et al. (2019) Italy
**Associated factors**	*Sexual minorities:* Psychosocial stress may represent an indirect pathway through which structural stigma contributes to mortality.	Hatzenbuehler et al. (2014) USA
**Theme 2: Stigmatisation, discrimination, victimisation and social exclusion as mental health challenges**		
	*LGBT individuals:* Discrimination, victimisation and social exclusion lead to social isolation (strong social resources through the life course may alleviate the negative impact of adverse experiences on health) and health disparities. Social and structural determinants contribute to sexual orientation-based mental health disparities, rather than the view that sexual orientation, itself, causes poor mental health. Stigmatisation, victimisation and familial rejection are linked to stress, depression, substance use and suicidality. Discrimination (homophobic), violence and low-community connectedness is positively related to depressive symptoms and alcohol use. Stigmatisation, discrimination and violence related to sexual-and gender-minority status across the life course. *Transgender individuals:* Distress and all types of dysfunction strongly predicted by experiences of social rejection and violence. Distress related to gender identity is very common (83%). Average level of distress is quite high (79·9 on a scale of 0 [none at all] to 100 [extreme]). Most (90%) experienced moderate family/social/work/scholastic dysfunction related to gender identity. *LGBTI individuals:* Discrimination (abuse and public insults, healthcare system (pathologising LGBTI and generic mainstream services) and social exclusion based on stigma, heterosexism, heteronormativity, homophobia and transphobia result in the following: -Fear of stigma and discrimination. -Not accessing preventative or responsive mental health services, or delaying access. -Up to 14 times more suicidal attempts amongst LGBTI people, rates of depression over five times higher amongst trans people and 3.5 times higher amongst LGB people than in general population. Discrimination (social, religious, employment and healthcare) and violence play a significant role in mental health; more vulnerable to mental healthcare problems than heterosexual–cisgender people.	Fredriksen-Goldsen et al. (2014) USA Ojanen et al. (2016) Thailand Lozano-Verduzco et al. (2017) Mexico Institute of Medicine (2011) USA Robles et al. (2016) Mexico Rosenstreich (2011) Australia Utama (2017) Indonesia
**Stigmatisation as a mental health challenge**	**Stigma and health inequalities** Stigma is a fundamental cause of health inequalities that influences mental health outcomes through multiple mechanisms. Stigma disrupts/ inhibits access to structural, interpersonal and psychological resources that could otherwise be used to avoid or minimise poor health.	Hatzenbuehler et al. (2013) USA
	**Consequences of stigmatisation** *Sexual minority individuals:* Those who live in high structural stigma communities die sooner, are more likely to die by suicide and at younger ages (18 years earlier, on average) versus low-stigma communities. *LGBT individuals:* Enacted stigma (explicit discrimination) may lead to discrimination in healthcare provision. Felt stigma (awareness of potential enacted stigma) may lead to delayed healthcare seeking based on fear. Internalised stigma (self-stigma) may lead to denigration of the self and feelings of not deserving the same access to healthcare as heterosexual people. Structural stigma (institutional stigma) may lead to disadvantaged and restricted opportunities. High reports of health-related stereotype threats significantly predicted high reports of fear of the physician, which could indicate fear to communicate with their providers on their mental health; slightly but significantly predicted delays in seeking mental health services. Health-related stereotype threats significantly predicted self-reported poor mental health outcomes. LGBT-related policy statements used stigmatising terminology and perpetuated anti-LGBT prejudice.	Hatzenbuehler et al. (2014) USA Institute of Medicine (2011) USA Ojeda-Leitner and Lewis (2019) USA Ojanen et al. (2016) Thailand
**Victimisation as a mental health challenge**	*LGBT youth:* Experience bullying and harassment at school. Experience elevated levels of violence, victimisation and harassment compared with heterosexual and non-gender-variant youth. *Identified four classes of victimisation trajectories:* Class 1 (65.4%): low, decreasing victimisation. Class 2 (10.3%): moderate, increasing victimisation. Class 3 (5.1%): high, steady victimisation. Class 4 (19.2%): high, decreasing victimisation. Youths in classes 2 and 3 were at higher risk of depression than those in class 1; youths in classes 2, 3 and 4 were at elevated risk of post-traumatic stress disorder. *LGBT individuals:* High and average levels of violence were positively associated with depressive symptoms and alcohol use; the greater the violence, the more likely the depressive symptoms and alcohol use.	Mallory et al. (2017) USA, Texas Institute of Medicine (2011) USA Mustanski et al. (2016) USA Lozano-Verduzco et al. (2017) Mexico
**Discrimination as a mental health challenge**	*Transgender and bisexual individuals:* Higher rates of unmet need and untreated depression are partly explained by differences in social factors, including experiences of discrimination, lower levels of social support and systemic exclusion from healthcare. Transgender individuals: Higher odds of reported discrimination, depression symptoms and attempted suicides compared with non-transgender individuals. Lack of self-acceptance of LGBT identity associated with depression symptoms. Discrimination: unfair treatment by employers, bosses and supervisors because of LGBT status and verbal insults or abuse. *LGBT individuals:* Experience discrimination in employment, housing and public accommodations that leads to economic instability. Experience health disparities. Average levels of discrimination (families, schools and healthcare systems) positively associated with alcohol use and aspects of mental health.	Steele et al. (2017) Canada. Su et al. (2016) USA Mallory et al. (2017) Texas, USA Lozano-Verduzco et al. (2017) Mexico
**Theme 3: Barriers to accessing mental health services as mental health challenges**		
**Sub-themes**	**Key results**	**Sources**
**Incidence of unmet access**	*Transgender individuals:* 1.6 times more likely to report untreated depression; 2.4 times as likely to report an unmet need for mental healthcare as cisgender heterosexual women. *Bisexual individuals:* 1.8 times as likely to report an unmet need for mental healthcare as cisgender heterosexual women.	Steele et al. (2017) Canada.
**Barriers to access**	*LGBT individuals:* Generic factors (overcrowding, stigma and confidentiality concerns). Anticipation of practitioners not being accepting or understanding LGBT identities. Low practitioner knowledge of LGBT issues. Stereotyping of LGBT clients. Long waiting lists Being diagnosed and being LGBT Stigma and discrimination Lack of treatment choices and concerns about treatment choices – medication and the ‘right therapist’	Ojanen et al. (2016) Thailand Rutherford et al. (2012) Canada McCann and Sharek (2014) Ireland
**Suggestions to improve access**	*LGBT individuals:* ‘Responsive, seamless, and holistic services’. Being treated with dignity and respect. Ideas for curbing stigmatisation and discrimination. Mental health practitioners to be better educated about LGBT issues. Good practice guidelines. Psychoeducation for support at work and for significant others. Develop a practice focus on LGBT mental health. Need for LGBT-sensitive mental healthcare services. Develop LGBT mental health training programmes.	McCann and Sharek (2014) Ireland Rutherford et al. (2012) Canada

LGBTQ, lesbian, gay, bisexual, transgender and queer; LGBTI, lesbian, gay, bisexual, transgender and intersexual; LGBT, lesbian, gay, bisexual and transgender; LGB, lesbian, gay and bisexual.

### Ethical consideration

This article followed all ethical standards for a research without direct contact with human or animal subjects.

## Results

This section discusses the key results of the literature review under the following themes: (1) emotional distress as a mental health challenge; (2) stigmatisation, discrimination, victimisation and social exclusion as mental health challenges; and (3) barriers to accessing mental healthcare services as a mental health challenge. The research populations used in the reviewed publications are indicated by different acronyms, such as LGB or LGBT.

### Theme 1: Emotional distress as a mental health challenge

Emotional distress reported in the studies included adverse childhood experiences, depression, anxiety, suicidal ideation and attempts. The minority stress theory is used to explain the effects of the unique stressors experienced by LGBT individuals.

Blosnich and Andersen (2015:3) mentioned that LGB individuals reported significantly higher mean totals of adverse childhood experiences than their heterosexual peers. These pre-existing stressors, such as sexual abuse, physical abuse and peer victimisation, may exacerbate the poorer mental health outcomes of LGB people compared with heterosexual individuals. With regards to specific mental health problems, the consensus study of the Institute of Medicine (2011:4) found that LGB youth are at increased risk of suicidal ideation and attempts and depression, whilst LGB adults appear to experience more depressive and anxiety disorders and suicidal ideation and behaviour than heterosexual adults. Little research has examined the prevalence of depressive and anxiety disorders amongst transgender people (Institute of Medicine 2011:233). A literature review similarly indicated that LGBT populations are vulnerable to higher rates of depression and suicidality in the midst of facing numerous mental health disparities (Mongelli et al. 2019:47). According to Kerr, Santurri and Peters (2013:185), bisexual women and lesbians had a greater likelihood of having mental health issues and used significantly more mental healthcare services than heterosexual women. The higher utilisation was ascribed to respondents who had free access to campus mental healthcare services. Bisexual women reported the worst mental health status with anxiety, anger, depressive symptoms, self-injury, and suicidal ideation and attempts. Up to 14 times more suicidal attempts were reported amongst LGBTI people, and rates of depression were over five times higher amongst transgender people and 3.5 times higher amongst LGB people than in the general population (Rosenstreich 2011:16–18).

In addition to emotional distress, five studies explored the minority stress theory. According to the theory, LGB individuals experience unique social stressors, including victimisation and discrimination, as a result of their minority position. These stressors trigger related internal stress that has negative effects on the health of LGB individuals (Mongelli et al. 2019:28). Internal stress includes experiences, such as homophobia, expectations of rejection and identity concealment (Fredriksen-Goldsen et al. 2014). Attempts to conceal LGBTQ status, prejudice and internalisation of negative societal views led to stress, self-isolation, lowered self-esteem and negative mental health outcomes in LGBTQ individuals (Heck 2015:3–4; Mongelli et al. 2019:28). There is a need for studies to explore intrapersonal strengths or coping strategies as ways to overcome minority stress (Russell & Fish 2016:9).

Hatzenbuehler et al. (2014:9–10) suggested that the psychosocial stress experienced by sexual minorities may represent an indirect pathway through which structural stigma contributes to mortality. This is illustrated by a rise in cardiovascular disease-related deaths in high-stigma communities compared with low-stigma communities.

### Theme 2: Stigmatisation, discrimination, victimisation and social exclusion as mental health challenges

Stigmatisation, discrimination and victimisation emerged as the most evident determinants of mental health problems, whilst different forms of social exclusion were also mentioned in some studies. Some publications investigated only one of these determinants, whilst others explored the combined effects of two or more determinants. The latter studies are presented in this section, followed by a discussion of each determinant as a sub-theme.

According to Fredriksen-Goldsen et al. (2014:8), it is not so much sexual orientation, itself, that causes mental health problems, but rather social and structural determinants that contribute to sexual orientation-based mental health disparities. The stigmatisation, discrimination and violence that LGBT individuals suffer during their lifetime related to their sexual and gender minority status undeniably affect their mental health status (Institute of Medicine 2011:5; Utama 2017:25–26). A historical overview of LGBT identities and issues in Thailand linked to stress, depression, substance use and suicidality to stigmatisation, victimisation and familial rejection (Ojanen, Ratanashevorn & Boonkerd 2016:41). Similar results by Lozano-Verduzco, Fernández-Niño and Baruch-Domínguez (2017:224) linked depressive symptoms and alcohol use to homophobic discrimination, violence and low-community connectedness towards LGBT communities in Mexico.

Distress and dysfunction (family, social, work or scholastic) were strongly predicted in transgender individuals who experienced social rejection and violence (Robles et al. 2016:856). The emotional distress caused by heterosexism, heteronormativity, homophobia, transphobia and stigma is further exacerbated by fears of stigma and discrimination that hinder access to mental healthcare services (Rosenstreich 2011:16–18).

#### Stigmatisation as a mental health challenge

This sub-theme explains the types and effects of stigma, as well as pathways through which stigma perpetuates health disparities in LGBT individuals.

Stigma is defined as ‘the co-occurrence of labeling, stereotyping, separation, status loss, and discrimination in a context in which power is exercised’ (Hatzenbuehler, Phelan & Link 2013:813). Stigma presents itself in different ways. Overtly expressed, enacted stigma takes the form of explicit behaviours, such as labelling, discrimination and violence, targeting people because of their perceived gender nonconformity (Institute of Medicine 2011:62). Covert stigmatisation includes, for example, LGBT-related policy statements that used stigmatising terminology and perpetuated anti-LGBT prejudice in Thailand (Ojanen et al. 2016:41). Stigmatisation may have devastating effects. LGBT individuals who live in communities with greater prejudicial attitudes against sexual minorities die sooner than those who live in communities with low levels of structural stigma (Hatzenbuehler et al. 2014:9).

Stigma restricts health access to LGBT individuals, be it structural stigma based on institutional processes or felt stigma based on an internal awareness that the potential for stigma exists in a specific situation (Institute of Medicine 2011:64). Two pathways are suggested through which stigma perpetuates health disparities in LGBT individuals. Firstly, LGBT people who experience internalised stigma (self-stigma), which leads to the denigration of the self, may feel that they do not deserve respect from healthcare providers or the same access to healthcare as heterosexual people. As a result, they may not disclose relevant information to healthcare providers or may avoid seeking treatment (Institute of Medicine 2011:64). Secondly, stereotypes attached to LGBT individuals within the healthcare services (also known as health-related stereotype threats) lead to fear of communicating with providers about mental health and delays in seeking mental healthcare services. These threats significantly predicted self-reported poor mental health outcomes in LGBT individuals (Ojeda-Leitner & Lewis 2019:9–10).

It is clear from the above information that stigmatisation of sexual minorities may disrupt or inhibit access to structural, interpersonal and psychological resources otherwise available to avoid or minimise poor health conditions (Hatzenbuehler et al. 2013:819).

#### Victimisation as a mental health challenge

The publications focused on how different forms of victimisation inter-relate with mental health consequences in LGBT individuals.

Victimisation against LGBT individuals takes many forms, such as harassment, bullying and elevated levels of violence (Institute of Medicine 2011:42; Mallory et al. 2017:37 & 43). Mustanski, Andrews and Puckett (2016:531) found that 10.3% of LGBT youths in Chicago experienced significant increases in victimisation and 5.1% maintained high levels across time, placing LGBT youths at risk for depression and post-traumatic stress disorder. High and average levels of violence were positively associated with depressive symptoms and alcohol use in LGBT individuals in Mexico; the greater the violence, the more likely the depressive symptoms and alcohol use (Lozano-Verduzco et al. 2017:224).

Utama (2017:26) illustrated the way victimisation of LGBTI people in Indonesia interrelates with other social determinants to increase the risk of mental illness. Some LGBTI people are disconnected from their families because of rejection that manifests itself in physical and psychological violence. Some decided to leave school because of exposure to peer group violence, leaving them at risk for unemployment. In some cases, employers terminated the services of gay and lesbian people because of their sexual orientation. Feeling victimised, ostracised, and without social and financial security and support, these individuals are at risk for mental health problems (Utama 2017:26).

#### Discrimination as a mental health challenge

Four studies in the review indicated the correlation between discrimination against LGBT persons at personal, familial and societal levels and mental health problems.

In Texas, discrimination that denies LGBT people equal access to essential social structures, such as housing, public accommodation and employment, not only led to economic instability and lack of productivity but also rendered them vulnerable to health and mental health disparities (Mallory et al. 2017:3–6). A Canadian study (Steele et al. 2017:120) measured the effects of discrimination based on unfair treatment by employers, bosses and supervisors because of LGBT status and verbal insults or abuse. Experiences of discrimination, lower levels of social support and systemic exclusion from healthcare partly explained higher rates of unmet needs and untreated depression in transgender and bisexual individuals. Transgender individuals reported higher odds of reported discrimination, depressive symptoms and suicidal attempts compared with non-transgender individuals.

A lack of self-acceptance of LGBT identity was associated with depressive symptoms in a US study (Su et al. 2016:19). Even average levels of discrimination from families, schools and the healthcare system were positively associated with alcohol use and aspects of mental health in LGBT individuals (Lozano-Verduzco et al. 2017:224).

### Theme 3: Barriers to mental healthcare services as a mental health challenge

Four studies indicated how barriers to accessing mental healthcare services contributed to unmet healthcare needs amongst LGBT people.

The mental health disparities that LGBT individuals suffer are explained by differences in social factors, discrimination, lower levels of social support and systemic exclusion from healthcare services (Steele et al. 2017:120). Transgender women in Canada were 1.6 times more likely to report untreated depression and 2.4 times as likely to report an unmet need for mental healthcare compared with cisgender heterosexual women. Bisexual women were 1.8 times as likely to report an unmet need for mental healthcare compared with cisgender heterosexual women (Steele et al. 2017:120).

Two qualitative studies explored LGBT-specific barriers to accessing mental healthcare services. Mental healthcare providers highlighted low practitioner knowledge of LGBT issues and the stereotyping of LGBT clients as barriers (Rutherford et al. 2012:908). The LGBT individuals in the study by McCann and Sharek (2014:4–6) experienced subtle ways of stigma and discrimination, such as being overlooked (‘not having a voice’) and being stereotyped during a consultation. They were concerned about getting the ‘right therapist’ (a therapist sensitive towards LGBT issues) and ‘being diagnosed and being LGBT’, referring to concerns that being LGBT is linked to mental health issues.

The overview of mental healthcare services in Thailand distinguished between client-related barriers (anticipation that practitioners may not be accepting or understanding of LGBT identities) and practitioner-related barriers (stereotyping remarks during the consultation) (Ojanen et al. 2016:50).

Lesbian, gay, bisexual and transgender individuals made suggestions to improve their access to mental healthcare services. They wished for ‘responsive, seamless, and holistic services’ and being treated with dignity and respect. Some ideas for curbing stigma and discrimination included good practice guidelines and training for mental health practitioners on LGBT issues. They also wanted mental healthcare practitioners to provide psychoeducation at their workplaces and for significant others (McCann & Sharek 2014:5–7). Rutherford et al. (2012:907) recommended the development of a practice focus on LGBT mental health, LGBT-sensitive mental healthcare services and LGBT mental health training programmes.

## Discussion

Lesbian, gay, bisexual and transgender individuals experience higher levels of emotional distress. The minority stress model (Meyer 2003:35) helps to understand the relationships between (1) external stressors such as stigmatisation, discrimination and victimisation based on a person’s minority status and (2) internal stressors such as expectations of rejection, concealment of sexual orientation and internalised homophobia. The internal distress may lead to negative mental health outcomes, whilst coping strategies and social support may counteract these negative outcomes. Additional emotional distress may relate to adverse childhood experiences, but research is recommended to understand why LGB individuals are more likely to report childhood victimisation (Blosnich & Andersen 2015:3).

Fredriksen-Goldsen et al. (2014) presented the Health Equity Promotion Model to stimulate more inclusive LGBT research. This model highlights (1) heterogeneity and intersectionality within LGBT communities; (2) the influence of structural and environmental context; and (3) health-promoting and adverse pathways that encompass behavioural, social, psychological and biological processes. Social status, for example, marginalisation, and social isolation may lead to negative self-worth that is linked to internalisation of discrimination. Social support, however, fosters resilience to withstand the negative effects of external stressors. Psychological processes include effective ways of coping, such as problem-solving, opposed to ineffective ways of coping, such as avoidance (Fredriksen-Goldsen et al. 2014:7–10; Mayock et al. 2009:137). Whilst LGBT individuals share collective experiences of stigmatisation and discrimination, experiences of oppression may vary across subgroups, leading to different mental health outcomes (Smalley, Warren & Barefoot (2016:100).

Interpreted within the results of this review, the models link negative external processes (stigmatisation, discrimination and victimisation) to negative internal processes (emotional distress). Mental healthcare services are well-positioned to assist LGBT individuals to mobilise positive external processes (social support) and to develop positive internal processes (effective coping strategies, resilience and self-worth). However, LGBT people still experience barriers to accessing mental health services and are frequently ‘invisible’ to healthcare providers and researchers. Overcoming this invisibility in healthcare services and research settings are critical to eliminating health disparities (Institute of Medicine 2011:14). An assessment of the evidence in the United Kingdom (Semlyen, Johnson & Barnes 2018:4) identified not only effective services that offer LGBT-specific interventions but also significant gaps in service provision and knowledge. More research is needed to develop culturally appropriate models of care for LGBT people. Bidell (2016:9) highlighted structural barriers such as lack of sensitive, affirmative and competent clinical services, as well as practitioner-related barriers where the provider’s personal beliefs conflict with professional LGBT ethical standards. Stigma remains to be an important barrier, as well as LGBT clients choose not to identify themselves as LGBT (Smith et al. 2019:202).

Whilst competent and ethical LGBT mental healthcare services and professional training may help to address the mentioned barriers, more innovative methods such as self-reflection and self-awareness are required to address practitioners’ personal beliefs (Bidell 2016:9). Providers should focus on the creation of a safe, non-judgmental environment to help clients realise that they will not face discrimination if they identify themselves (Smith et al. 2019:202).

## Implications and recommendations

The review has implications for healthcare and mental healthcare providers alike. Healthcare providers need to take note of the results and treat LGBT people with sensitivity and respect so that they may feel free to access healthcare services and raise their mental health concerns without fearing discrimination, victimisation and stigmatisation. The LGBT people with signs and symptoms of emotional distress ought to be referred to mental healthcare providers for psychosocial interventions in order to prevent the development of psychiatric disorders. Psychological interventions are essential to facilitate the development of effective coping strategies and resilience. Social interventions should focus on two levels: firstly, family interventions to facilitate acceptance and support, and secondly, advocacy for and participation in right-based and empowering policy initiatives related to the LGBT population. As said by Fredriksen-Goldsen et al. (2014:11), the achievement of health equity requires empowering LGBT people to take action and address the environmental and structural barriers that influence their health.

Whilst the evidence from this review indicates both provider-based mental healthcare disparities and perceived disparities based on LGBT individuals’ expectations, further research is recommended to explore the inter-relationship between different types of disparities to provide possible interventions.

The mental healthcare needs of minority populations and subsequent treatment implications should be included in the curricula of healthcare providers. Inservice training using reflective techniques may help to facilitate mental healthcare providers’ awareness of their own beliefs and stereotypes that may hinder effective management of LGBT individuals.

## Limitations

The researchers used the acronym LGBT as a search term, thereby overlooking literature referring to other sexual minority subgroups. The results reflect the collective mental health challenges experienced by the LGBT community, and not so much the between-group differences within this community. By following this approach, the review fell short of providing a more in-depth understanding of each subgroup’s individual mental health needs.

## Conclusion

The results showed that LGBT communities still experience significant emotional distress and mental health challenges as a result of stigmatisation, victimisation, discrimination and barriers to accessing mental healthcare services. Specifically, LGBT youth still experience a magnitude of mental health problems, and there are few empirically supported approaches for working with LGBT youth in clinical settings (Russell & Fish 2016:15). If healthcare providers acknowledge and apply their ethical duty to treat all people with respect and dignity, this can help to relieve the mental health disparities of LGBT people. Future studies need to explore how mental healthcare providers can support LGBT individuals to develop resilience and challenge social discourses that maintain discriminatory and stigmatising practices, most of all in mental healthcare services. To meet the diverse needs of the LGBT community, future reviews should explore and compare mental health challenges across different subgroups.
